# Genomic survey and expression analysis of *LcARFs* reveal multiple functions to somatic embryogenesis in *Liriodendron*

**DOI:** 10.1186/s12870-024-04765-7

**Published:** 2024-02-07

**Authors:** Lin Xu, Ye Liu, Jiaji Zhang, Weihuang Wu, Zhaodong Hao, Shichan He, Yiran Li, Jisen Shi, Jinhui Chen

**Affiliations:** Key Laboratory of Forest Genetics and Biotechnology, Ministry of Education of China, Co-Innovation Center for the Sustainable Forestry in Southern China, Nanjing Forestry University, No.159 Longpan Road, Nanjing, 210037 China

**Keywords:** *ARF* genes, *Liriodendron chinense*, Somatic embryogenesis

## Abstract

**Background:**

Auxin response factors (ARFs) are critical transcription factors that mediate the auxin signaling pathway and are essential for regulating plant growth. However, there is a lack of understanding regarding the *ARF* gene family in *Liriodendron chinense*, a vital species in landscaping and economics. Thus, further research is needed to explore the roles of *ARFs* in *L. chinense* and their potential applications in plant development.

**Result:**

In this study, we have identified 20 *LcARF* genes that belong to three subfamilies in the genome of *L. chinense.* The analysis of their conserved domains, gene structure, and phylogeny suggests that *LcARFs* may be evolutionarily conserved and functionally similar to other plant *ARFs*. The expression of *LcARFs* varies in different tissues. Additionally, they are also involved in different developmental stages of somatic embryogenesis. Overexpression of *LcARF1*, *LcARF2a*, and *LcARF5* led to increased activity within callus. Additionally, our promoter-GFP fusion study indicated that *LcARF1* may play a role in embryogenesis. Overall, this study provides insights into the functions of *LcARFs* in plant development and embryogenesis, which could facilitate the improvement of somatic embryogenesis in *L. chinense*.

**Conclusion:**

The research findings presented in this study shed light on the regulatory roles of *LcARFs* in somatic embryogenesis in *L. chinense* and may aid in accelerating the breeding process of this tree species. By identifying the specific *LcARFs* involved in different stages of somatic embryogenesis, this study provides a basis for developing targeted breeding strategies aimed at optimizing somatic embryogenesis in *L. chinense*, which holds great potential for improving the growth and productivity of this economically important species.

**Supplementary Information:**

The online version contains supplementary material available at 10.1186/s12870-024-04765-7.

## Introduction

*Liriodendron chinense* is an angiosperm species with leaves resembling traditional Chinese garments and cup-shaped flowers, making it a significant ornamental, economic, and forestry tree [1–3]. Crossbreeding is a crucial method to improve the genetic diversity of *Liriodendron* and cultivate hybrids with superb forest characteristics [4]. However, its low natural seed setting rate, difficult rooting during cutting propagation, and low survival rate of grafting breeding impede this species' popularization and application. Somatic embryogenesis, a cost-saving procedure, can be employed to propagate hybrid plants while maintaining their superior traits [5–7]. Somatic embryogenesis for *Liriodendron* involves a multi-step regeneration process, including embryonic callus induction, somatic embryo induction and maturation, and plantlet germination [5, 7]. In order to successfully generate somatic embryos, it is crucial to regulate the development of the embryo, particularly the transition of cell fate [8, 9].

Plant hormones have a significant influence on somatic embryogenesis. Among them, auxin plays the most crucial role in acquiring embryogenic potential [10]. It is achieved by controlling gene expression via *ARFs*, which bind to cis-elements in downstream genes by utilizing two distinct DNA-binding domains [11]. *ARF* genes encode auxin response transcription factors that bind to TGTCTC auxin responsive elements in the promoters of early auxin response genes. A typical ARF protein contains several functional domains: a plant-specific DNA-binding domain (DBD) at the N-terminus, transcriptional activation or repression domain in the middle region (MR), and a C-terminal dimerization domain (CTD) in most cases [12]. The DBD has a B3-type DBD followed by dimerization domains (DD). DD can cause *ARFs* to form dimers, essential for binding to target DNA [11]. The CTD is related to motifs III and IV found in Aux/IAA (Auxin /Indole-3-Acetic Acid) proteins [13]. *Aux / IAA* family members can form dimers with *ARFs* via the CTD domain [14]. *ARFs* bind to *Aux/IAA*, which inhibits its activation on auxin responsive genes at low auxin concentrations. Under high auxin levels, auxin acts as a molecular glue between the TIR1/AFB (Transport Inhibitor Resistant 1/Auxin Signaling F-box) receptor and Aux/IAA protein, leading to subsequent ubiquitination and degradation of Aux/IAA protein, releasing *ARFs* from inhibition. Therefore, *ARFs* are crucial in the auxin regulatory pathway by converting chemical signals into gene regulation [15].

ARF proteins play important roles in various aspects of plant growth and development, including the development of roots, flowers, and embryogenesis. In *Arabidopsis*, *AtARF7* and *AtARF19* control lateral root formation through interaction with three IAA proteins (IAA3, IAA14, IAA18) [16]. *AtARF6* and *AtARF8* regulate petal expansion, stamen filament elongation, anther dehiscence, and pistil maturation, which ensure that pollen released from anthers is deposited on the stigma of the recipient [17]. In the early stages of zygotic embryogenesis, auxin is transferred from basal cells to apical cells, which induces embryo development. In later stages, the direction of auxin flux is reversed, leading to auxin accumulation in the hypothesis and triggering the onset of root meristems [18]. Many *ARFs* are expressed and involved in zygotic embryogenesis. At the spherical embryo stage in *Arabidopsis*, *AtARF1* is ubiquitous at the globular stage, *AtARF5* marks the lower tier of the embryo, but *AtARF2*/*9* are expressed in all suspensor cells and the lower-tier of protoderm cells [19]. At the heart embryo stage, *AtARF1*/*2* are ubiquitously expressed, and *AtARF5* is active in subdomains of the vascular tissue. *AtARF9* is expressed in the presumptive root meristem [19]. *AtARF9*, redundantly with *AtARF13*, mediates the differentiation of suspensor cells and prevents its transition to the embryo [20]. *AtARF5/7* interact with each other physically and control both axis formation in the embryo and auxin-dependent cell expansion [21, 22].

Some researches reveal that *ARFs* also play the same role during somatic embryogenesis as in zygotic embryogenesis. Extensive expression of many *AtARFs* in *Arabidopsis* embryogenic culture suggests that auxin signaling may play an important role in somatic embryogenesis induction [23]. In rice, down-regulation of *OsARF5*, a positive regulator of stem cell maintenance and meristem development, may account for better somatic embryo regeneration and differentiation in japonica than in indica [24]. Some studies in *Arabidopsis* have indicated that *AtARFs* are involved in acquiring embryonic competence in somatic embryogenesis [23]. *Liriodendron* is a noteworthy species in the fields of horticulture and economics. However, traditional breeding methods have proven to be time-consuming and inadequate in meeting the demands of current production. To address this issue, we have employed somatic embryogenesis as a breeding strategy to achieve rapid plant propagation. In this process, auxin serves as a crucial factor in regulating the efficiency of somatic embryogenesis. Isolating and identifying relevant genes, as well as thoroughly investigating the expression patterns of target genes during somatic embryogenesis, will greatly enhance the efficiency of this process and expedite the breeding of *Liriodendron*. Therefore, our study aims to isolate an important auxin signal transcription factor - auxin response factor (*ARF*) - from the entire genome of *Liriodendron*. The isolation and identification of this gene family are crucial for elucidating the mechanism underlying somatic embryogenesis in *Liriodendron*.

## Results

### Genome-wide identification of *ARFs *in *L. chinense*

**Fig. 1:**
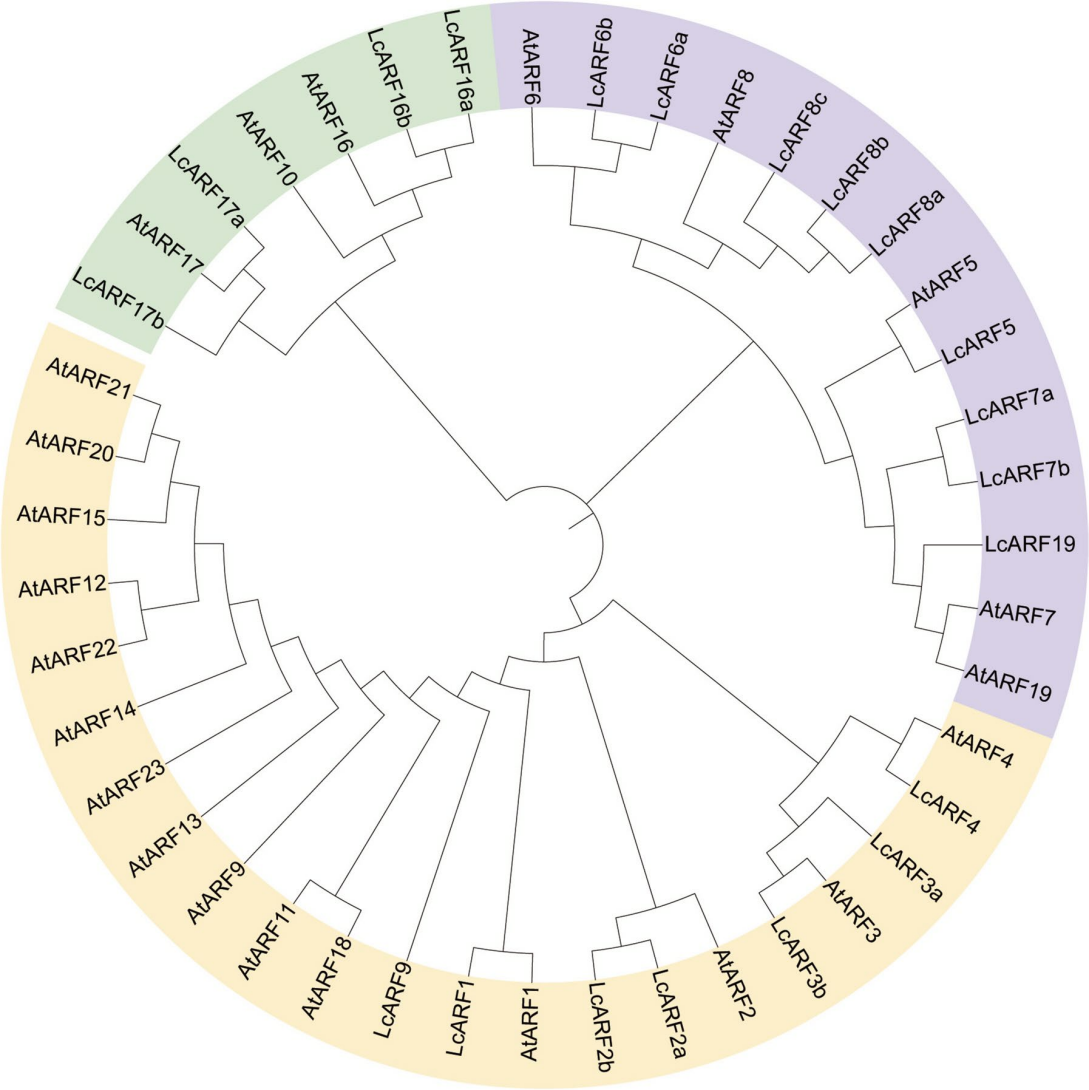
Phylogenetic tree of ARF proteins in *Arabidopsis* and *L. chinense*. The ARFs can be classified into three major classes based on their phylogenetic relationship. The different-colored areas represent distinct classes within the ARF family

ARF protein sequences from *Arabidopsis* as reference, 20 candidate *ARFs* were identified in *L. chinense* genome by BLASTP and HMMER programs (see methods). The *LcARF* members were named based on the homology with *AtARFs* in this study (Fig. 1 and Table 1). The molecular weight of these LcARFs was between 47.46 kDa and 133.46 kDa. The theoretical PIs of these LcARFs were 4.6 ~ 9.13 (Table 1), indicating that most *LcARFs* encoded weakly acidic proteins. All *LcARFs* were predicted to localize in nucleus (Table 1).

**Table 1:** Physicochemical properties and chromosomal details of LcARF proteins

Gene ID	Gene name	Number of amino acids	Molecular weight	Theoretical pI	Formula	Total number of atoms	Instability index	Aliphatic index	Grand average of hydropathicity	Predicted location(s)
Lchi01654	LcARF1	590	65956.41	5.69	C_2901_H_4506_N_814_O_888_S_30_	9139	55.89	70.54	-0.469	Nucleus.
Lchi16424	LcARF2a	740	82677.75	5.64	C_3610_H_5599_N_1023_O_1129_S_40_	11401	56.69	59.03	-0.716	Nucleus.
Lchi02396	LcARF2b	763	85164.6	6.03	C_3746_H_5823_N_1061_O_1157_S_29_	11816	58.86	65.52	-0.64	Nucleus.
Lchi09892	LcARF3a	597	65843.48	4.86	C_2911_H_4450_N_790_O_912_S_23_	9086	45.95	73.79	-0.394	Nucleus.
Lchi08511	LcARF3b	683	75266.67	6.32	C_3346_H_5146_N_930_O_1008_S_23_	10453	57.66	71.79	-0.451	Nucleus.
Lchi19395	LcARF4	731	81623.34	4.6	C_3505_H_5451_N_997_O_1170_S_42_	11165	47.58	66.94	-0.662	Nucleus.
Lchi29014	LcARF5	424	47755.46	8.96	C_2134_H_3307_N_599_O_614_S_18_	6672	52.87	77.03	-0.333	Nucleus.
Lchi18918	LcARF6a	928	102934.55	6.14	C_4538_H_7045_N_1293_O_1389_S_31_	14296	67.57	78.36	-0.419	Nucleus.
Lchi18919	LcARF6b	314	35268.28	8.84	C_1569_H_2444_N_446_O_452_S_15_	4926	62.55	77.68	-0.321	Nucleus.
Lchi10785	LcARF7a	1193	133463.24	9.13	C_5837_H_9272_N_1722_O_1770_S_49_	18650	64.17	77.42	-0.566	Nucleus.
Lchi05212	LcARF7b	902	98651	5.52	C_4315_H_6760_N_1216_O_1352_S_42_	13685	56.4	75.04	-0.375	Nucleus.
Lchi12475	LcARF8a	601	66621.31	5.84	C_2966_H_4598_N_814_O_893_S_21_	9292	56.29	80.42	-0.301	Nucleus.
Lchi12473	LcARF8b	601	66693.38	5.76	C_2969_H_4602_N_814_O_895_S_21_	9301	56.95	80.42	-0.306	Nucleus.
Lchi28544	LcARF8c	452	49572.42	4.94	C_2176_H_3359_N_601_O_687_S_20_	6843	49.87	72.08	-0.309	Nucleus.
Lchi09822	LcARF9	744	83171.29	7.96	C_3662_H_5779_N_1051_O_1107_S_29_	11628	57.78	73.64	-0.512	Nucleus.
Lchi08159	LcARF16a	692	75991.06	7.52	C_3343_H_5235_N_947_O_1015_S_33_	10573	48.01	73.11	-0.368	Nucleus.
Lchi05394	LcARF16b	582	64144.49	6.1	C_2816_H_4412_N_802_O_861_S_27_	8918	51.13	74.4	-0.368	Nucleus.
Lchi33157	LcARF17a	572	62416.55	8.26	C_2780_H_4282_N_776_O_823_S_21_	8682	47.11	68.99	-0.295	Nucleus.
Lchi20149	LcARF17b	445	48464.55	5.41	C_2137_H_3314_N_578_O_668_S_21_	6718	45.92	67.87	-0.421	Nucleus.
Lchi11431	LcARF19	570	64248.68	6.34	C_2853_H_4412_N_796_O_851_S_24_	8936	56.53	75.26	-0.394	Nucleus.

19 *LcARFs* were distributed on 11 chromosomes of *L. chinense*, except *LcARF17a* was mapped to a separate scaffold not yet assembled into full chromosomes (Fig. S1). There were 4 *LcARFs* mapped on chromosome 3, which contains most of the *LcARFs*. The rest *LcARFs* per chromosome varied from 0 to 4, with no apparent correlation between chromosomal length and the number of *LcARFs* present.

One way for organisms to acquire new genes is gene duplication. We found that a few *LcARFs* had extremely high sequence similarities (Fig. 1). *LcARF2a-2b*, *LcARF3a-3b*, *LcARF6a-6b*, *LcARF7a-7b*, *LcARF16a-16b* and *LcARF17a-17b* form twin pairs, and *LcARF8a/b/c* from triplet pairs. Surprisingly, no tandem duplication events were found because most of the duplicated *LcARFs* were located on different chromosomes, or they are separated by at least a few million bases if on one chromosome (Table 1).

### Phylogenetic analysis of *LcARFs*

**Fig. 2:**
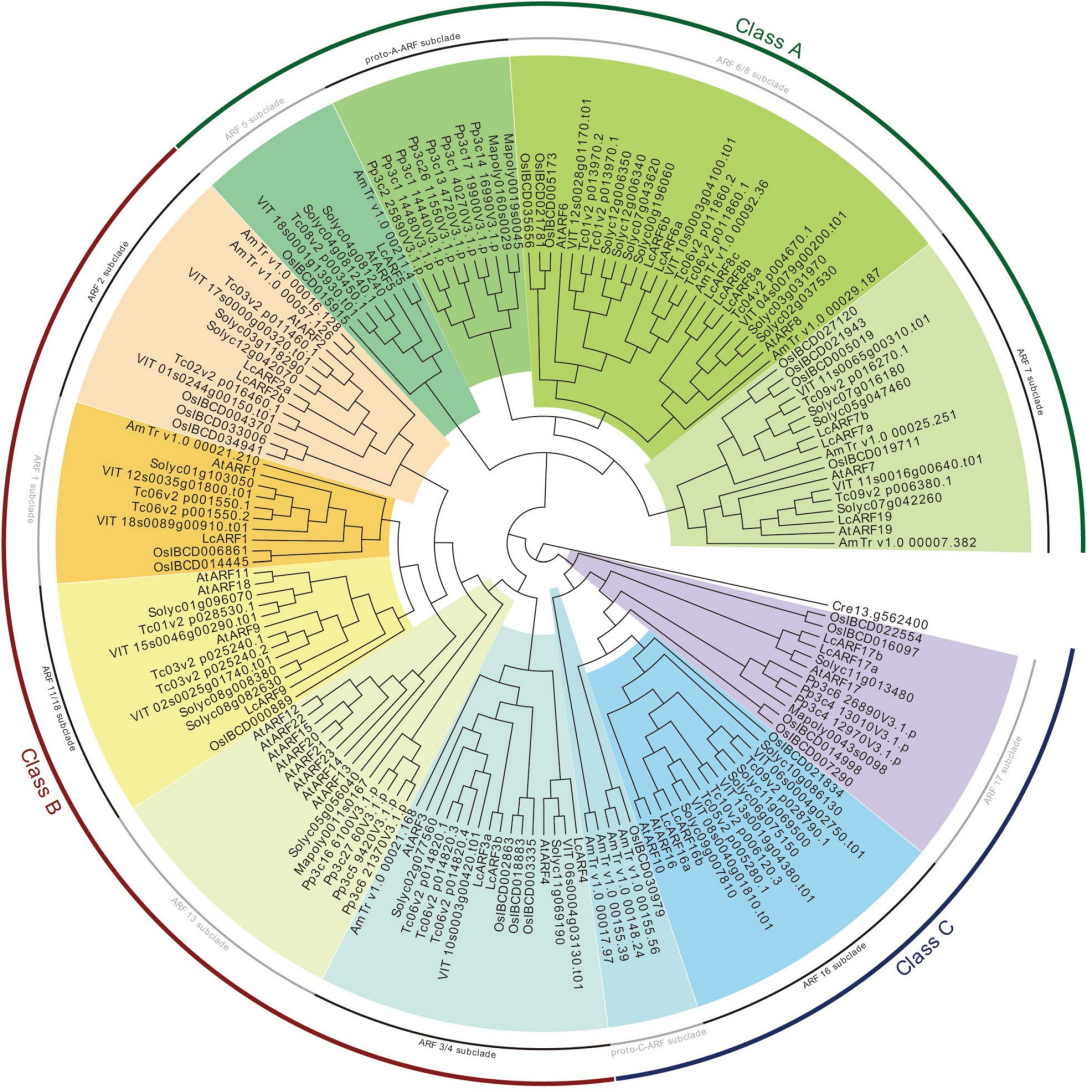
Unrooted Classification tree representing relationships among *ARF* genes of 10 species. A total of 162 ARF protein sequences from 10 species were selected to construct a Bayesian phylogenetic tree. Different color blocks represent different evolutionary branches

We expanded the neighbor-joining analysis to include *ARF* sequences from other taxa to gain insight into the evolutionary relationship between *LcARFs* and their homologs in other plant species. Including typical species of algae, bryophytes, herbs, woody plants, monocots, dicots, etc.; 4 ARF sequences from the liverwort *Marchantia polymorpha*, 15 sequences from the moss *Physcomitrella patens*, 23 sequences from rice, 17 sequences from *Vitis vinifera*, 21 sequences from *Theobroma cacao*, 25 sequences from *Solanum Lycopersicum*, 13 sequences from *Amborella trichopoda* and 23 from *Arabidopsis* (Fig. 2). A B3 domain-containing sequence from green algae *Chlamydomonas reinhardtii* was used as an outgroup. In this analysis, 162 ARF sequences were included in a moderately well-supported phylogenetic tree. Cre13.g562400 from *C. reinhardtii* was the most divergent gene as it showed sequence similarity to *ARF* genes in the B3 DNA-binding region and lackeds other motifs. Consistent with analyses by Mutte et al. (2018) and Finet et al. (2013), the *ARFs* can be classified into three major clades on their phylogenetic relationship; class A, class B, and class C (Fig. 2).

We further analyzed the phylogeny of the *ARF* family in more detail (Fig. 2). Class A contained four subclades, and the guide branch percentages well supported the relationship among the subclades in clade A. ARF5 subclade, ARF6/8 subclade and ARF7 subclade are the A-ARFs in land plants, while contains the proto-A-ARF subclade in bryophytes. Node e is the foundation of the other four subsections, indicating that they may have very conserved domains representing the A-ARF class precursors in plants. Branch B can be further divided into 6 subclades, including node f (ARF11 / 18 subclade), g (ARF13 subclade), h (ARF1 subclade), i (ARF2 subclade), j (ARF3/4 subclade), and k (bryophytes B-ARF). Remarkably, node k was the foundation of the five subsections: f, g, h, i, and j, indicating that they may represent precursors of the B-ARF class in land plants. Node g subsection contains all members of the *Arabidopsis* ARF family, which indicates that ARF may be repeated and diversified in eukaryotic plants. Branch C consists of node l (ARF16 subclade), m (bryophytes C-ARF), and n (ARF17 subclade). As observed in clades A and B, we found that the node m daughter clade was only represented by bryophyte members, meaning they may be a group of proto-C-ARF.

### Conserved domain analysis of LcARFs

**Fig. 3:**
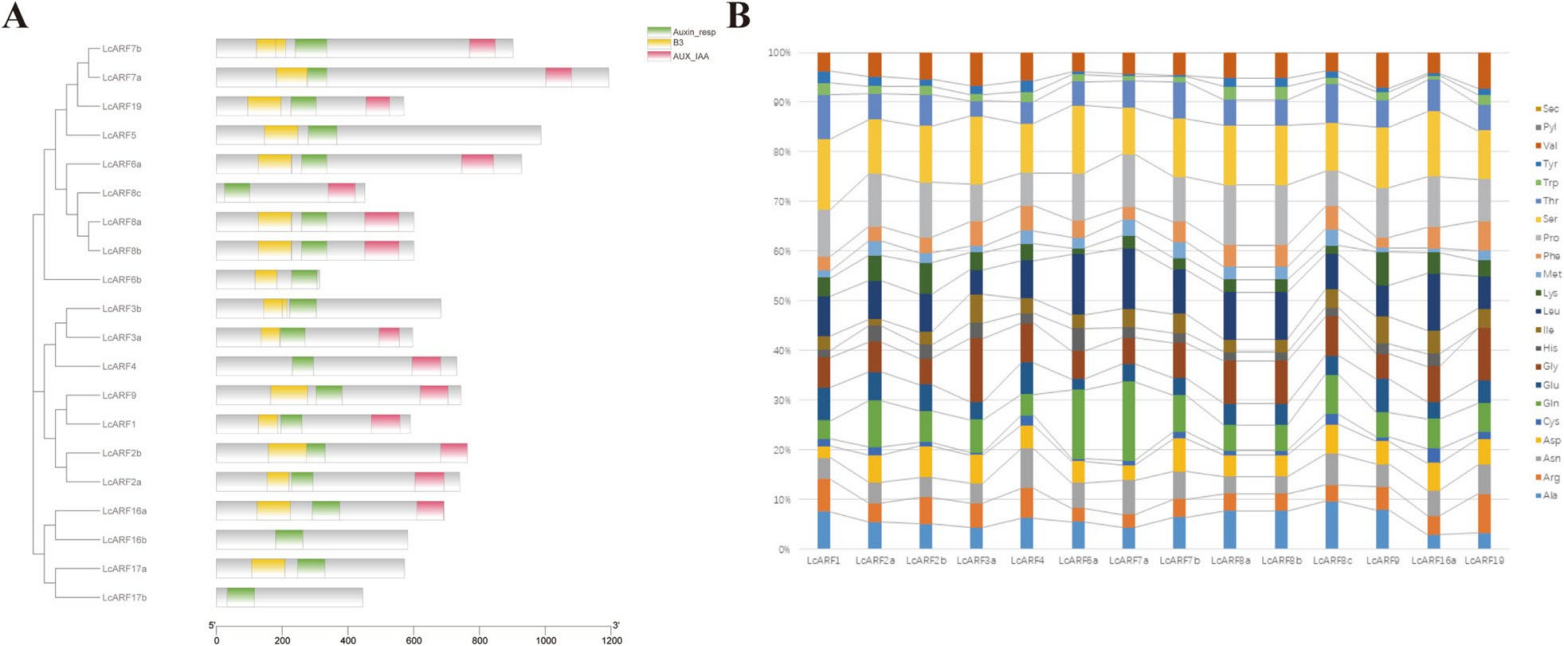
Analysis of conserved domains of *LcARF* gene family. **A** Schematic organization of conserved domains in LcARF proteins. **B** Amino acid composition of MR domains in LcARF proteins, bars represent the percentage of different amino acid residues in MR domains of LcARFs

Most ARF proteins contain a conserved N-terminal DNA-binding domain (DBD) composed of plant-specific B3-type and auxin responsive motifs, and a highly conserved C-terminal CTD domain corresponding to motif III and IV of the Aux/IAA proteins. To better understand the structural similarity of these ARFs, we constructed a neighbor-joining phylogenetic tree using the amino acids sequences of 20 LcARFs, resulting in 3 major *ARF* classes, subclade A-C (Figs. 3A, S2). The results demonstrated that LcARF4, LcARF8c, LcARF16b, and LcARF17b do not have a DBD domain. It is possible that the DNA-binding ability of these proteins could be impaired due to lack of a DNA-binding domain. The remaining LcARFs contained highly conserved and complete DBD domains. In addition, most LcARFs had conserved and intact CTD domains, except for LcARF3b, LcARF5, LcARF6b, LcARF16b and LcARF17a/b. Due to the lack of the AUX_IAA binding domain, the expression of these proteins may not be regulated by AUX/IAA.

Unlike the DBD and CTD regions are conserved, the protein sequences of the MRs (the middle region ) are highly variable. Six LcARFs not only lacked AUX_IAA domain, but also l MR domain (Fig. 3A). It has been proposed that ARF proteins whose middle regions are enriched in Ser, Pro, Thr, and Gly, might act as transcriptional repressors, whereas those are enriched in Gln and Leu in MR might act as transcriptional activators. The MRs of LcARF2a, LcARF6a, and LcARF7a were abundant in Gln and Leu residues, suggesting they might be transcriptional activators (Fig. 3B). Ser, Pro, Thr, and Gly were abundant in the MRs of LcARF1, LcARF8a and LcARF8b, suggesting that they might be transcriptional repressors (Fig. 3B). In summary, we speculated that the conserved domains of LcARFs were beneficial to ensuring the execution of its essential functions. In contrast, the diversity of MR domains helped LcARFs respond to different environments' adaptability.

### Gene structural analysis of *LcARFs*

**Fig. 4:**
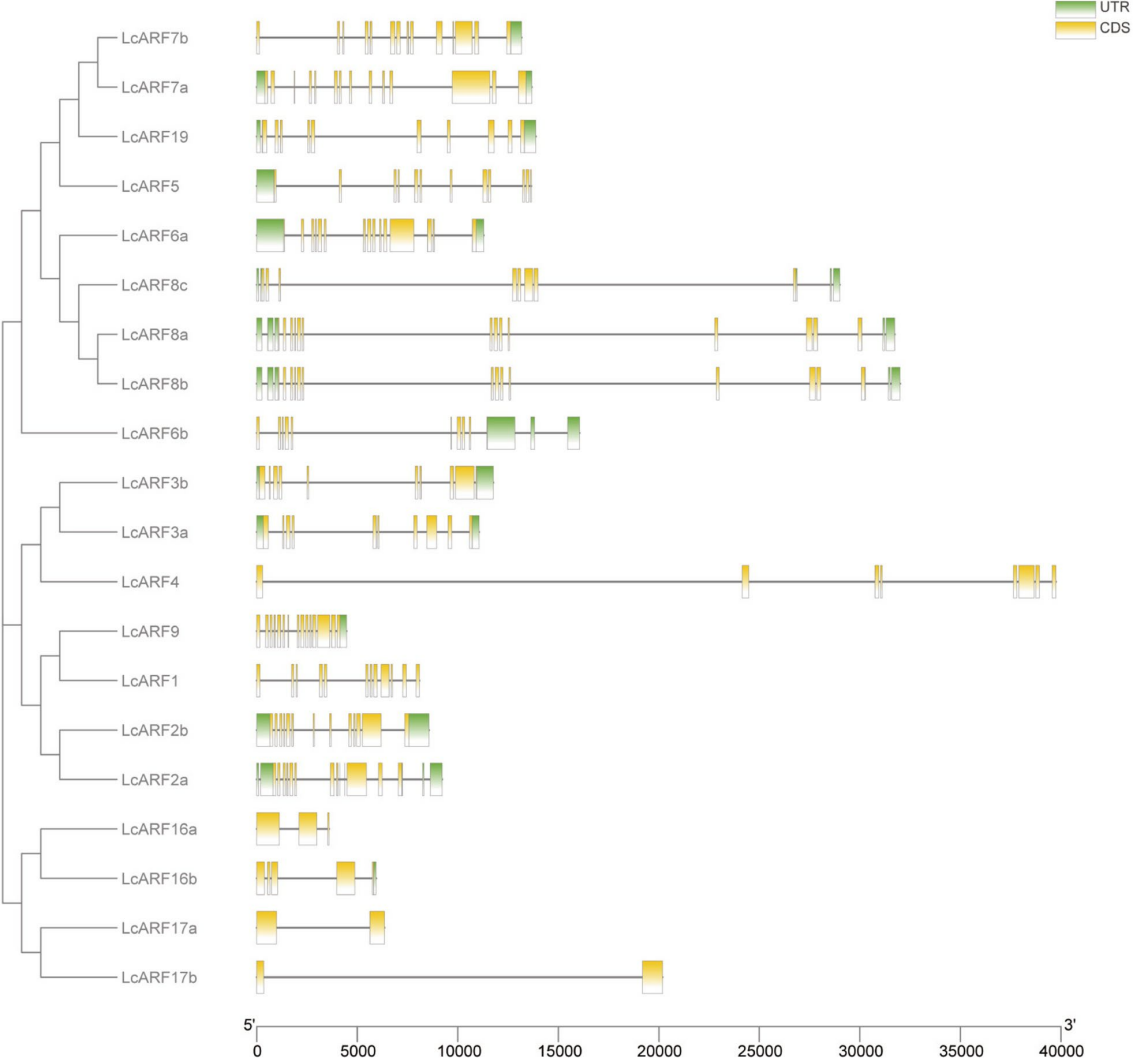
Analysis of intron-exon organization of *LcARF* gene family. The intron-exon organization of *LcARF* genes was plotted using Tbtools (Version 1.09832)

In addition to conserved protein domains, the pattern of intron-exon positions between ARF subgroups can also provide clues on evolutionary relationships. To identify the intron-exon structure of individual *LcARFs*, an alignment of the full-length cDNA sequences with the corresponding genomic DNA sequences was performed. For the members in the *LcARF1/2a/2b/5/6a/7a/7b/8a/8b/9* clade had 11~15 introns, members of *LcARF3a/3b/4/6b/8c/19* clade had 7~10 introns, and members of *LcARF16a/16b/17a/17b* clade had 1~4 introns (Fig. 4). Consistent with previous findings from *Arabidopsis* and rice, members of *LcARF16/17* clade had a relatively lower number of introns. Strikingly, the exon-intron structure was conserved among subclades of homologous genes and differed between subclades of non-homologous genes. The phylogenetic analysis of LcARF proteins also supported this.

### Numbers of cis-acting elements in *LcARFs*

**Fig. 5:**
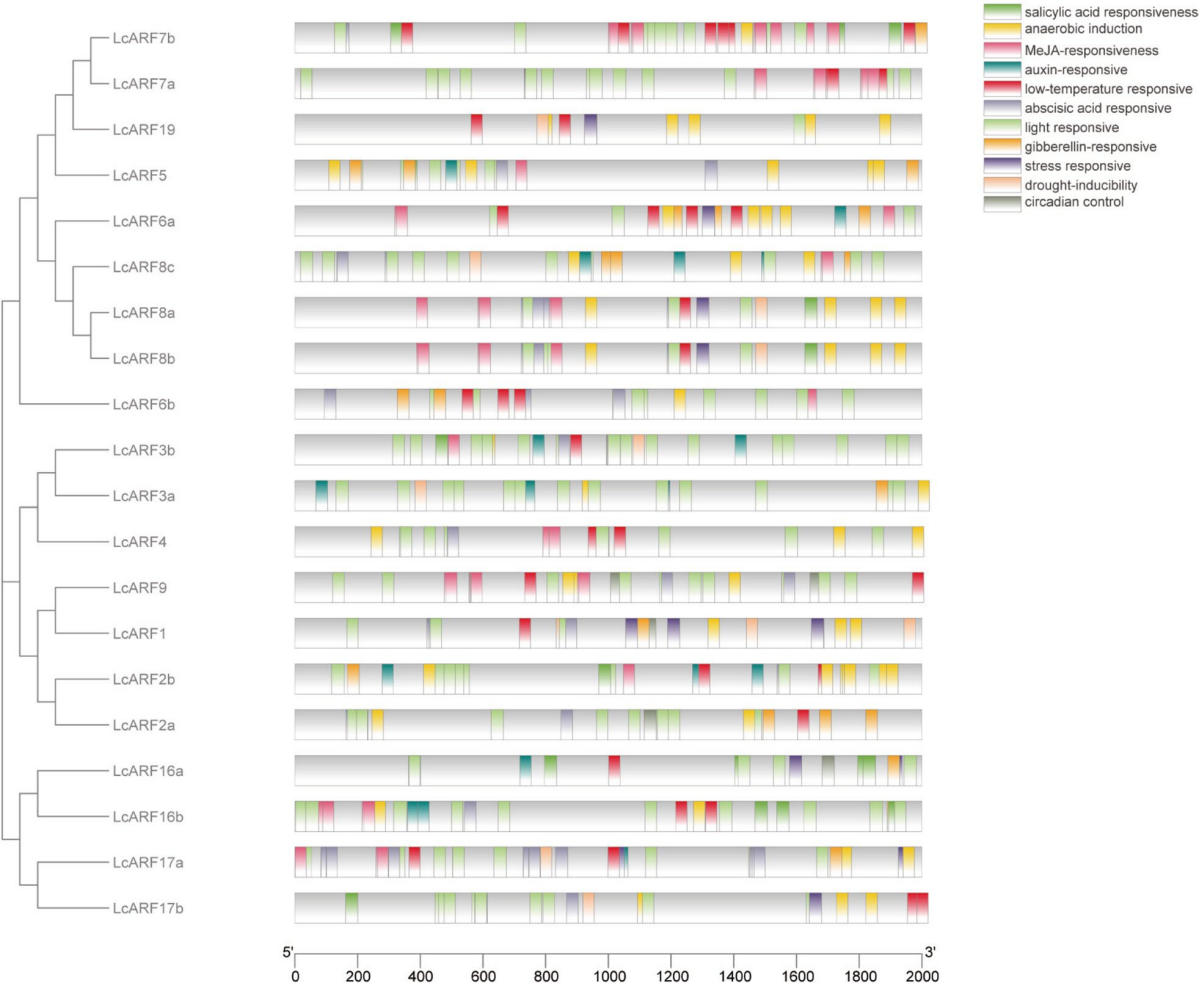
Analysis of cis-acting elements of *LcARF* gene family. The 2000-bp regulatory region upstream of ATG was analyzed with the PlantCARE software

To study the potential functions of *LcARFs* in *L. chinense*, we use the 2000 bp sequence upstream of ATG (start codon) to search cis-acting elements in the analysis of *LcARFs*. There were 11 types of cis-elements in these upstream regions, such as light response, temperature response, stress response, and others (Fig. 5). Almost all *LcARFs* had hormone response elements. *LcARF2a/7b/8a/8b/9/16b/17a/17b* had the most ABA-responsive elements. *LcARF4/6a/7a/7b/8a/8b/9/16b/17a* had most MeJA-responsive elements. *LcARF1* also had more drought response elements than other elements. Furthermore, *LcARF6a/6b/7a/7b/9* contained low-temperature response elements. It suggested that hormones or stress can influence the expression of *LcARFs*. All *LcARFs* had light response elements, revealing that these genes may play an important role in responding to light. Moreover, *LcARFs* also contained circadian elements related to plant growth and development, indicating that *LcARFs* might be involved in plant growth and development.

### Interactions between LcARFs

**Fig. 6:**
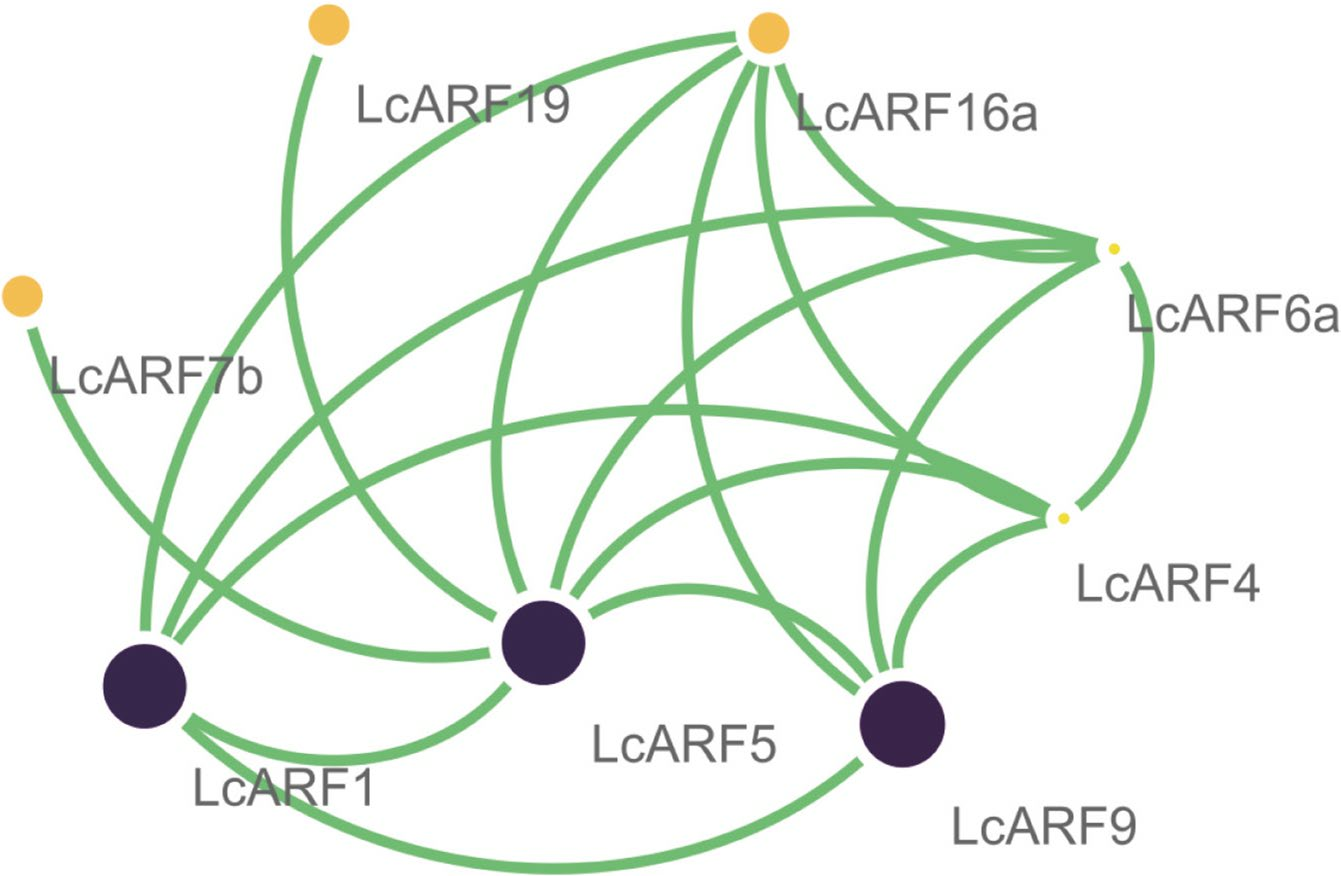
Prediction of protein interaction. Protein-protein interaction network of ARFs in *L. chinense*, the results were based on an *Arabidopsis* association model

In order to study the interaction between LcARFs to form homo/hetero-dimer, we predicted the protein-protein interaction network of LcARFs using *A. thaliana* as a reference (Fig. 6), and eight interacting links of LcARFs were found. It was conceivable that these interacting blocks of LcARFs in *L. chinense* may regulate different biological processes. Except for LcARF7b and LcARF19, the remaining 6 LcARFs interact with each other.

### Subcellular localization of LcARFs

**Fig. 7:**
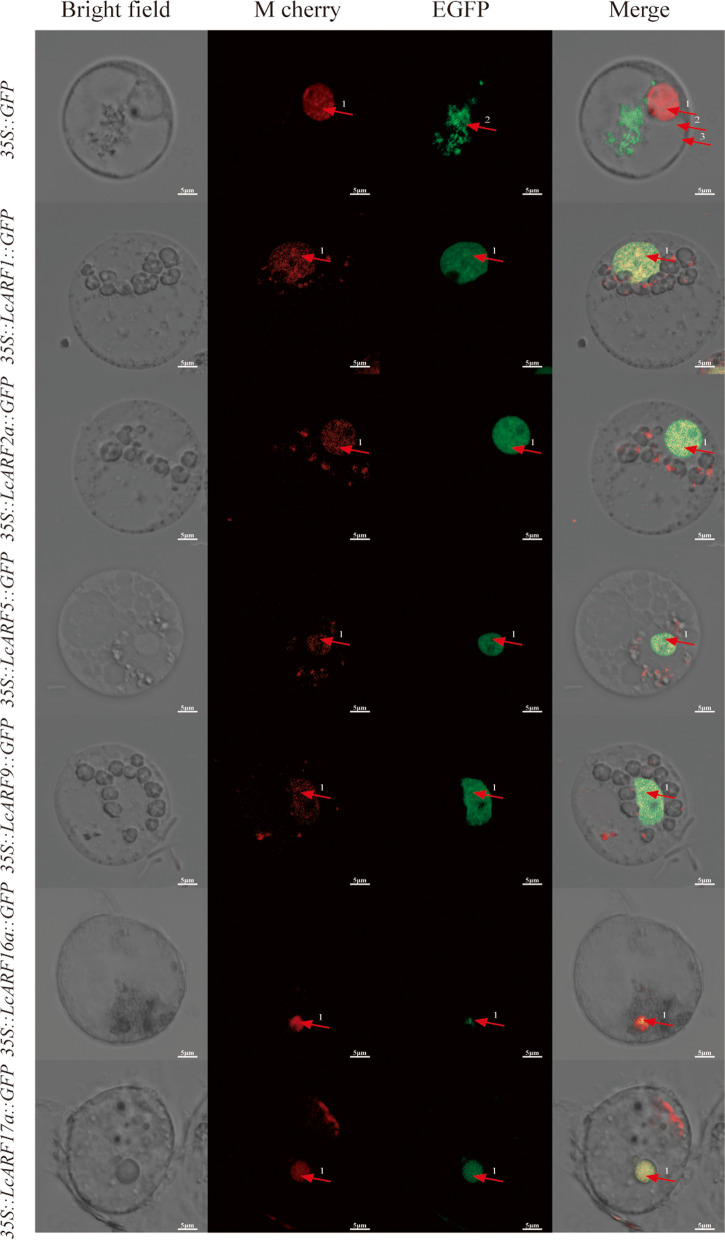
Subcellular localization of LcARF in *L. chinense* protoplasts. The red fluorescence signal was the nuclear localization signal of H2B, and the green fluorescent signals of the six GFP fusion proteins were particularly strong in the nucleus

The location of the 20 LcARFs was all predicted to be nucleus localization. To verify the results, we select six *ARFs* were selected for the transient expression in protoplasts from the callus of *L. chinense*. We used a 35S promoter to drive coding sequences of *ARFs* fused with GFP (*35S::ARF::GFP*). GFP was only detected in the nucleus, suggesting that six ARFs were located in the nucleus (Fig. 7), consistent with the predicted results.

### *LcARFs* tissue-specific expression

**Fig. 8:**
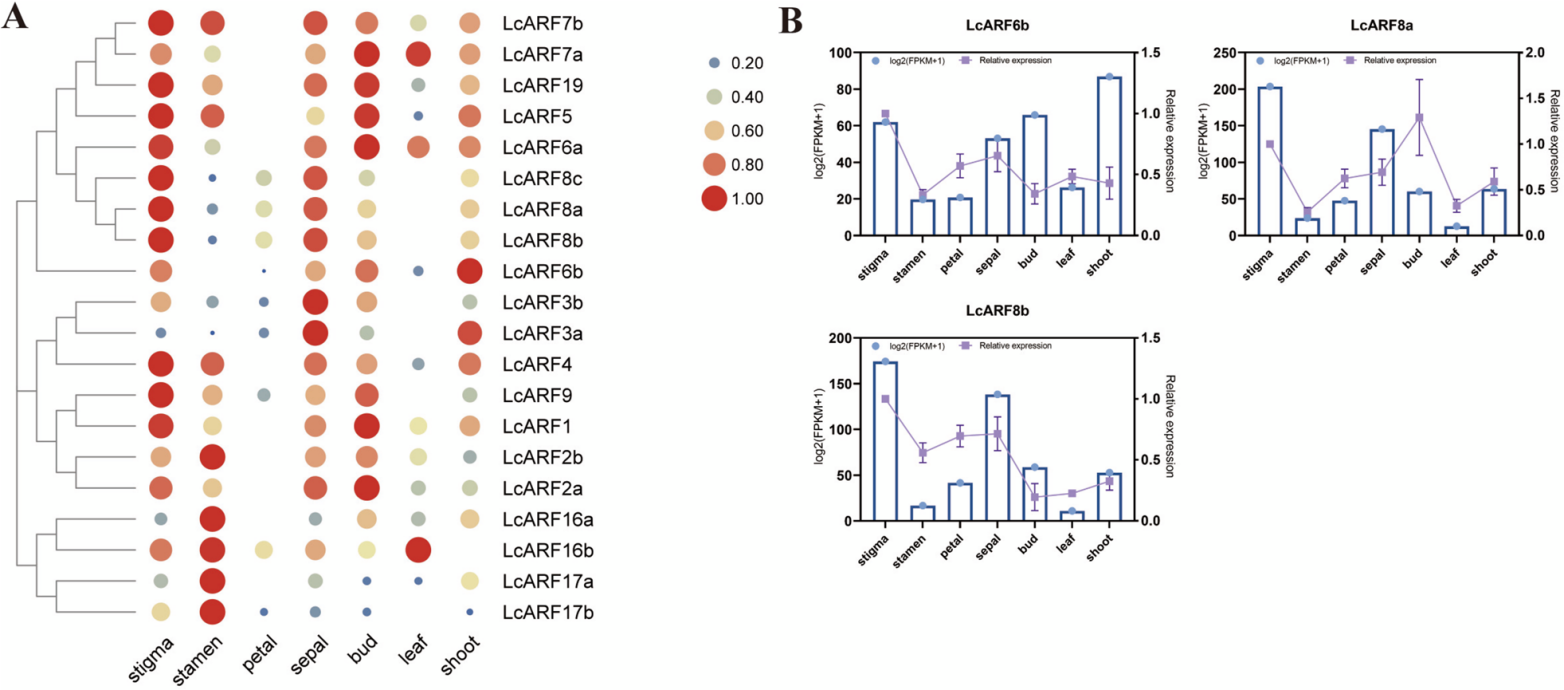
Expression patterns of *LcARFs* in different tissues, analyzed by qRT-PCR. **A** The expression pattern of *LcARFs* in different tissues. **B** qRT-PCR was used to detect the expression pattern of *LcARFs* in different tissues. The purple line graph represents the results of qRT-PCR experiments, with the scale on the right ordinate of each graph. The blue histogram represents the results of FPKM analysis, with the scale on the left ordinate of each graph

To understand how *LcARFs* might act during *L. chinense*’ growth development, we profiled 7 transcriptomes of different organs/tissues (shoot, leaf, bud, stigma, stamen, sepal, petal) using RNA-seq to quantify the expression patterns of all 20 *LcARFs* (Fig. 8A). We found that all 20 *LcARFs* showed tissue-specific expression. Class A and B genes were highly expressed in stigma, sepal, and bud. All members of class C were expressed explicitly in the stamen. These results indicated that *LcARFs* were mainly expressed in young tissues, and their functions may be mainly related to flower development (such as stigma, stamen, or bud).

To validate transcriptome data from different tissues, we performed qRT-PCR experiments on three selected genes (Fig. 8B). Three genes had a high stigma and sepal expression levels with the same expression patterns. This result was consistent with RNA-seq (Fig. 8A). Overall, the expression of *LcARFs* was tissue-specific, and the specific expression in sexual organs and buds revealed that this gene family had essential functions in flower and bud development.

### *LcARFs* universally expressed at different stages of somatic embryogenesis

**Fig. 9:**
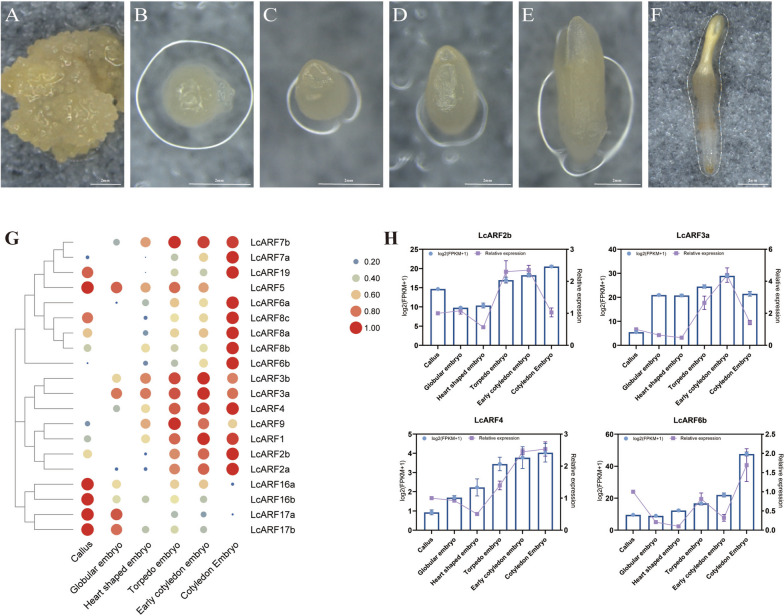
Expression analysis of *LcARFs* under SE, analyzed by qRT -PCR. **A** Callus. **B** Globular embryo. **C** Heart-shaped embryo. **D** Torpedo embryo. **E** Early cotyledonary embryo. **F** Cotyledon embryo. **G** The expression pattern of *LcARFs* in somatic embryos. **H** qRT -PCR was used to detect the expression pattern of *LcARFs* in different stages of SE. The purple line graph represents the results of qRT-PCR experiments, with the scale on the right ordinate of each graph. The blue histogram represents the results of FPKM analysis, with the scale on the left ordinate of each graph

To explore *LcARFs* expression during somatic embryogenesis, we analyzed in 6 successive stages (callus, globular embryo, heart-shaped embryo, torpedo embryo, early cotyledon embryo, cotyledon embryo) of *L. chinense* somatic embryogenesis by RNA-seq to quantify the expression patterns of all 20 *LcARF* genes (Fig. 9). All 20 *LcARFs* were expressed during somatic embryogenesis. Most members of class A were mainly expressed at the cotyledon stage. Members of class B were mainly expressed in torpedo, early cotyledon, and cotyledon stages. Members of class C were highly expressed in callus. Therefore, we speculated that members of class A and class B may be mainly involved in late somatic embryogenesis. The gene of class C may be involved in the maintenance of stem cells and early somatic embryogenesis.

Next, we selected four genes of class A member *LcARF6b* and class B member (*LcARF2b/3a/4*) for qRT-PCR verification (Fig. 9G). The results of qRT-PCR proved the reliability of transcriptome data, that is, class A members were mainly highly expressed in cotyledon embryo, and class B members were mainly highly expressed from torpedo embryo to cotyledon embryo. It also laid a theoretical foundation for improving the efficiency of somatic embryogenesis and propagating excellent *L. chinense* species.

### Overexpression of *LcARFs* led to increased cell activity in callus

**Fig. 10:**
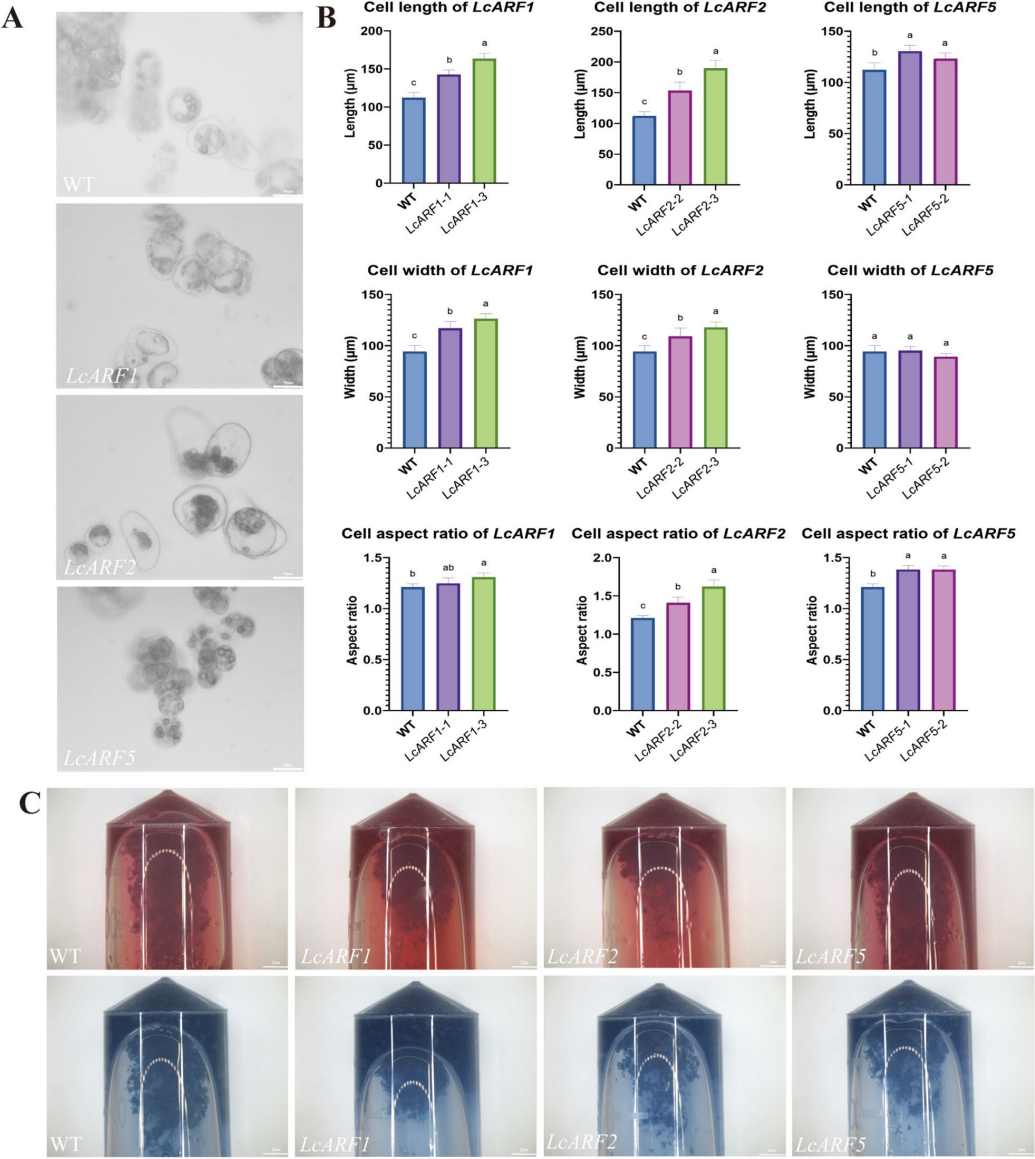
Phenotypic of calli overexpressing *LcARFs* in *Liriodendron*. **A** Cell morphology of calli from WT and overexpression lines. **B** Cell length, cell width, and cell aspect ratio statistics of calli from WT and overexpression lines. **C** Acetomagenta and Evans blue staining of calli from WT and overexpressed lines. *p* < 0.05

In our previous experiments focused on somatic embryogenesis, we discovered that the cell viability of callus directly impacted the efficiency of somatic embryogenesis. Based on transcriptome data analysis of *ARF*s in *L. chinense* at various stages of somatic embryo development, we selected *LcARF1/2a*, which had low expression levels in callus, and *LcARF5*, which had high expression levels, for further investigation. The 35S promoter was used to construct the overexpression vector, which was subsequently transferred into the callus of *Liriodendron* through genetic transformation. After conducting qRT-PCR experiments, we chose three overexpression lines for each genotype (Fig. S3). Our findings revealed that the cell area was notably larger in the transgenic lines overexpressing *LcARF2a* than in the wild type (Fig. 10A). Furthermore, additional measurements indicated that the cell length and width of the transgenic lines overexpressing *LcARF1/2a* were notably greater than those of the wild type (Fig. 10B). In addition, the cell aspect ratio of transgenic lines was substantially greater than that of the wild type, indicating that the overexpression of *LcARF1/2a/5* influenced the cell morphology of callus (Fig. 10B). Finally, staining the callus demonstrated that acetic acid magenta stained deeper in the overexpression lines. At the same time, Evans blue staining was lighter compared to the wild type (Fig. 10C). These results suggest that the overexpression of *LcARF1/2a/5* improves the cell viability of *L. chinense* callus.

### *LcARF1* may be involved in somatic embryogenesis

**Fig. 11:**
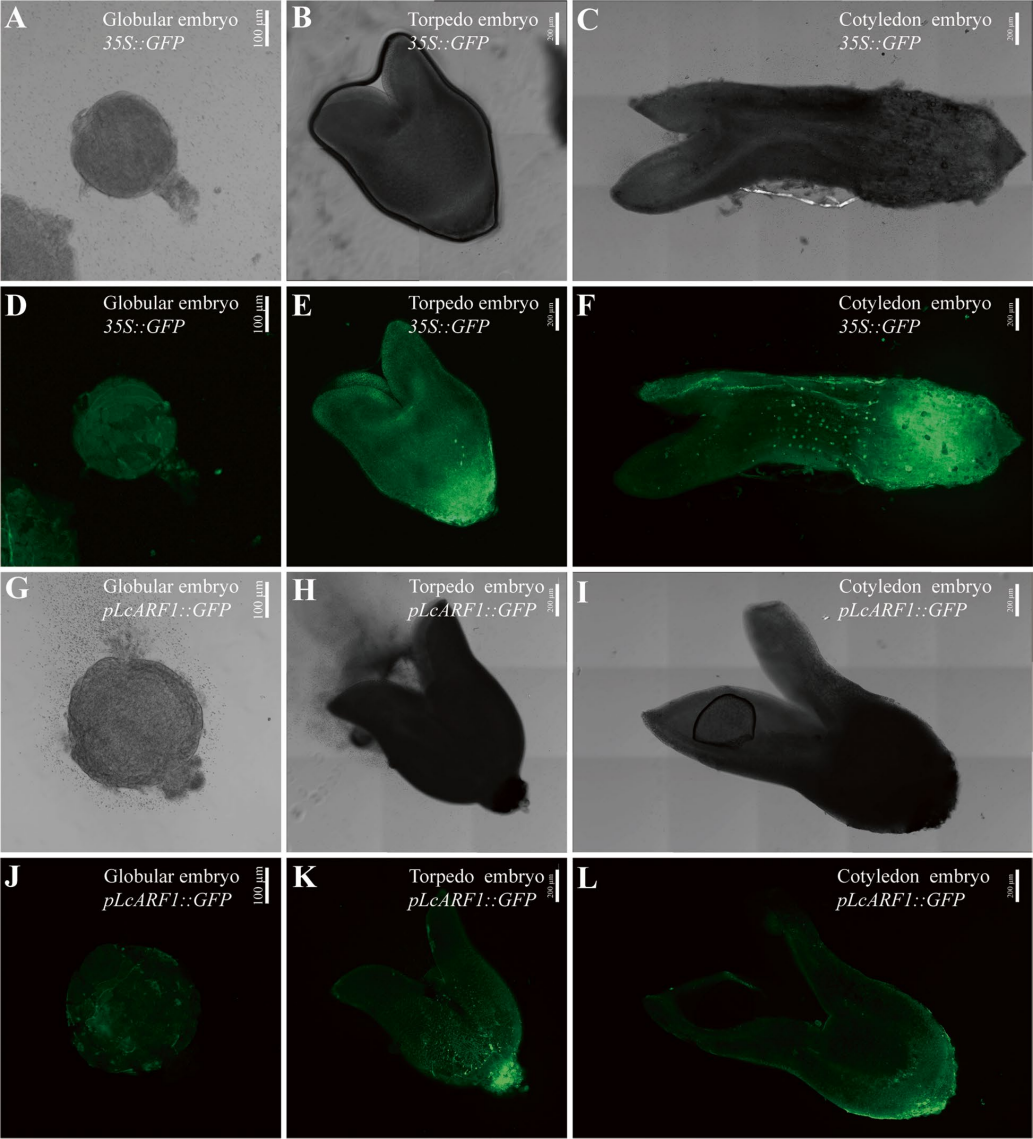
Expression pattern of *LcARF1* during somatic embryogenesis. **A**, **D**, **G**, **J**: Globular embryo. **B**, **E**, **H**, **K**: Torpedo embryo. **C**, **F**, **I**, **L**: Cotyledon embryos. **A**- **F**: 35S::GFP. **G**- **L**: *pLcARF1::GFP*

*ARF1* is widely expressed in the thallus of *Marchantia polymorpha*, while *ARF2* is mainly expressed in the marginal area around the thallus of *Marchantia polymorpha* [25]. In order to more intuitively understand the expression pattern of *LcARF* during somatic embryogenesis in *Liriodendron*, we constructed an *LcARF1* promoter-mediated GFP fluorescence signal vector and obtained transgenic lines (Fig. 11). Comparing it to the control group, we found that GFP triggered by the *LcARF1* promoter was primarily expressed in the morphological lower end of the torpedo embryo and morphological lower end of the cotyledon embryo (Fig. 11E, F, K, L). It suggests that *LcARF1* may be involved in regulating hypocotylation and root development. In the globular embryo stage, *pLcARF1*-induced fluorescence signals were distributed throughout the embryo, aligning with *Arabidopsis* report [19] (Fig. 11D, J).

## Discussion

### Molecular characterization and evolution of the *ARF* gene family in *L. chinense*

Auxin response factors are an essential group of signaling factors in the auxin signaling pathway [23, 26, 27]. They play a crucial role in regulating various physiological and developmental processes in plants by controlling the expression of downstream genes. *ARFs* are primarily involved in regulating plant hormone responses and biological responses to external stimuli [28]. *ARFs* were studied in many plants, including *Arabidopsis* [29], maize [30], rice [31], tomato [32], *Populus trichocarpa* [33], *Eucalyptus grandis* [34], apple [35], orange [36], physic nut [37], longan [38] and others. In this study, 20 *LcARFs* were identified in *L. chinense*. Given that the genome size of *L. chinense* was 1.75 Gb, nearly 12 times larger than the *Arabidopsis* genome [4], nearly 4 times larger than the rice genome [39], it was surprising that the number of *LcARFs* was less than that of *Arabidopsis* and rice. Apparently, our observations on the *ARF* gene family contradicted with genome complexity between *Arabidopsis*, rice, and *L. chinense*. Class B-ARFs contained 7 members in *L. chinense*, 9 members of rice, and 14 members in *Arabidopsis*. The number of *ARF* genes in *Arabidopsis* distributed in class-B is 2 times that of *L. chinense* and 1.56 times that of rice. Some researchers believed that several independent, small-scale, segmental replication events and chromosomal rearrangements had occurred at the *ARF13* locus, resulting in multiple members of the *AtARF13* family and ultimately the *AtARF* gene family in *Arabidopsis* Expansion [31]. In the evolution of angiosperms, whole-genome or segmental duplication and tandem duplication often occur, leading to the expansion of gene families [38]. In the study of rice and longan, researchers found that *ARFs* evolved through whole genome or segment duplication events, and there was no tandem duplication phenomenon [31, 38]. Our study confirmed that 20 *LcARFs* did not exhibit tandem duplication. It suggested that the number of members of the *LcARF* gene family may be associated with the loss of duplicated genes and the loss of tandem duplication events. Previous studies have shown that most of the *ARFs* of species such as *Arabidopsis* and rice have about six exons [31]. Our study found that the number of exons of *LcARFs* was much higher than that of rice and *Arabidopsis*, and most contained more than ten exons. It indicated that *LcARFs* might have more abundant functions.

Nuclear auxin signaling mediated by *ARF* transcription factors affects plant growth and development by regulating cell division, elongation, and differentiation. The evolutionary origin of the *ARF*-mediated pathway dates back to at least the common ancestor of bryophytes and other land plants [40, 41]. Because *L. chinense* is an ancient relic plant, in order to study the evolutionary trajectory of *LcARFs*, we conducted a comprehensive phylogenetic study using LcARF protein sequences from algae to terrestrial plants [42, 43]. Consistent with previous reports, LcARFs were composed of three main classes: class A (LcARF5/6/7/8), class B (LcARF1/2/3/4/9), and class C (LcARF10/16/17). In the three main LcARF*s* classes, we discovered all the bryophyte LcARF sequences, indicating that LcARFs may differentiate into three sets of transcription factors in the common ancestor of bryophytes, and this primordial event may be prior to the differentiation of liverwort and moss [42]. Surprisingly, in a phylogenetic analysis, we detected that these LcARF sequences formed three independent clusters based on each daughter class. Therefore, we speculate that the LcARF of these bryophytes may be identical to the counterpart of the ancestral *ARF* gene in land plants. The protein domain analysis found that all the 20 *LcARFs* contained Auxin_resp domain, consistent with previous studies, so *LcARFs* and other plants *ARFs* may be evolutionally conserved and functionally similar [44, 45].

### *LcARFs* may play significant roles in the growth and development of *L. chinense*

In recent years, the biological functions of *ARFs* in plant growth and development have been further studied. *ARF* transcription factors mediate the activity of the plant hormone auxin, regulating various aspects of plant development [46]. The researchers found that the *ARF* gene family was involved in the early development of cotton fibers and regulates early senescence in lilies and morning glories [47–49]. Specifically, *ARF* genes under class A were mainly related to shooting regeneration and adventitious root development. For example, in *Arabidopsis*, *AtARF5/7/19* played an important role in leaf vein development, *AtARF5* was involved in shoot regeneration, and *AtARF7/19* was involved in adventitious root development [50–52]. Class B branch genes were mostly related to the development of leaves, stems and roots. Studies on carrots, longan, corn and other species found that *ARF1/2* are involved in the growth and development of vegetative organs [38, 53, 54]. The *ARF* gene of class C is mainly related to gametophyte development. *ARF10/16/17* of grape and *ARF17* of tomato regulate parthenocarpy under the action of miR160 [55–57]. In addition, *AtARF2/4/5* was also involved in *Arabidopsis* gametophyte development [58]. In the reported literature, *ARF4* was a multifunctional gene, which was involved in gametophyte development and shoots regeneration in *Arabidopsis*, regulated the development of wheat roots and stems, and regulated the stomatal switch of tomato to enhance the response to salt damage and waterlogging [50, 59, 60]. In this study, we investigated the differential expression of *LcARFs* in tissues through transcriptome and qRT-PCR experiments. In accordance with findings from other species, classes A and B demonstrated high expression levels in vegetative organs. However, nearly all *ARFs* were found to be expressed in reproductive organs, with class A being predominantly expressed in stigma, class B exhibiting similar expression patterns in both stigma and stamens, and class C showing high expression levels mainly in stamens. This phenomenon may be attributed to the regulation of auxin during the development of male and female gametophytes. Overall, our findings suggest that *LcARF* may play a crucial role in the growth and development of *Liriodendron*, and the study of these key *LcARFs* may aid in the exploration and regulation of related functions at the molecular level. The results of these studies could further enhance the growth and reproduction rate of *Liriodendron*, potentially reducing the cost of wood utilization and promoting the use of afforestation applications.

### The role of *LcARFs* in somatic embryogenesis and their regulatory mechanisms

Somatic embryogenesis is induced by transcriptional reprogramming. Somatic cells respond to the induction signal and enter the embryonic development pathway after treatment with plant hormones, mainly auxin, to form somatic embryos [61]. Auxin triggers various molecular regulatory mechanisms during development, including *ARF*, the core component of the auxin signaling pathway [26, 62]. Signaling from *AtARF5* is necessary to form *Arabidopsis* callus shoots [63]. Mutations in *AtARF5* will result in severe patterning defects during embryonic and postembryonic development [64].

Somatic embryogenesis of *L. chinense* is a crucial method to obtain more seedlings. However, *LcARFs* regulating somatic embryogenesis have not been reported in *L. chinense*. Therefore, we explored the expression pattern of *LcARFs* during somatic embryogenesis. Expression trends of *LcARFs* in six stages of embryogenesis were divided into three groups. The first group was mainly class B members, with higher expression levels in torpedo embryos, early cotyledon embryos, and cotyledon embryos. The second group was mainly composed of class C members, which were highly expressed in callus and gradually decreased with the progress of somatic embryogenesis. The remaining *LcARFs*, the last group, were expressed at low levels during early somatic embryogenesis and high levels during the cotyledon embryonic stage. Interestingly, all *LcARFs* expression levels in globular and heart-shaped embryos were very low.As the vitality of callus, which is directly linked to the efficiency of somatic embryogenesis, is of utmost importance, it is critical to examine the effect of *ARFs* expression on callus condition. Based on the findings of transcriptome analysis, we selected *LcARF1/2a/5* for respective overexpression. The results indicated that the callus activity of the overexpression lines was greater than that of the wild type, suggesting that *ARFs* expression may influence or participate in the somatic embryogenesis process of *Liriodendron*. Utilizing the GFP fluorescence reporting system, we determined that *LcARF1* expression was present in the globular embryo, torpedo embryo and cotyledon embryo of *Liriodendron*. Moreover, we observed that *LcARF1* was mainly concentrated in the lower end of the embryo morphology. These findings suggest that *ARF1* actively participates in the somatic embryogenesis of *Liriodendron* and may potentially regulate root development.

In summary, according to previous research reports, *ARFs* have some essential functions in the process of somatic embryogenesis [65]. These results provide new clues for studying *ARF* genes involved in somatic embryogenesis. Therefore, *LcARFs* are worthy of further revealing their regulatory mechanism through molecular biology experiments. In the future, these important somatic embryogenesis regulatory genes will help improve the efficiency of the asexual reproduction of *L. chinense*, thereby increasing the reproductive ability of *L. chinense* and increasing the yield of trees.

## Conclusions

This study comprehensively identified the *Liriodendron chinense ARF* gene family. 20 *LcARFs* gene structures, conserved motifs, phylogeny, cis-acting elements, and protein interaction predictions were analyzed. Then, expression levels of *LcARFs* in different tissues and somatic embryogenesis were analyzed by RNA-seq and qRT-PCR simultaneously. We confirmed that the expression of *LcARFs* was tissue-specific and participated in the somatic embryogenesis process in *Liriodendron*. Furthermore, our study demonstrated that overexpressing *LcARF1/2a/5* enhances the activity of the callus, and we also established the involvement of *LcARF1* in the somatic embryogenesis process. The finding that *ARFs* expression plays a crucial role in the somatic embryogenesis of *Liriodendron* is an important step forward in understanding the breeding process of this species. These results have laid a strong foundation for the optimization of somatic embryogenesis conditions and the improvement of somatic embryo yields, ultimately meeting the needs of horticultural greening and industrial production.

## Materials and methods

### Datasets and sequence retrieval

The complete genome, transcript/protein sequences, and genome feature file of *L. chinense* were downloaded from https://db.cngb.org/search/project/CNP0000815/ [4]. All ARF proteins of *Arabidopsis* thaliana were obtained from the Plant Transcription Factor Database (http://planttfdb.gao-lab.org/). An ARF Hidden Markov profile (B3 DNA binding (Pfam 02362), Auxin_Resp (Pfam 06507) and AUX_IAA (Pfam 02309)) was retrieved from the Pfam website (http://pfam.xfam.org), and the protein sequences in the *L. chinense* genome were identified using HMMER (v.3.0.1b) and BLASTP. All identified ARFs were further validated by a conserved domain search using the conservative Domains Database (CDD; https://www.ncbi.nlm.nih.gov/Structure/cdd/wrpsb.cgi) and PFAM (http://pfam.xfam.org/) databases, whose E value cut of < 1E-5, consequently, the redundant and partial sequences were removed manually [66].

### Sequence analysis

For the gene structure illustration, we utilized the GFF3 file of the *L. chinense* genome and images were implemented by TBtools software [67]. The motifs analysis of ARF protein was performed by the Multiple Em of Motif Elicitation (MEME Suite) and then demonstrated by TBtools software. For each gene of *ARF*, the several physicochemical properties (i.e., molecular weight (MW), isoelectronic points (PIs), and others) were intended by ExPASY PROTPARAM tools (http://web.expasy.org/protparam/ ). Subcellular localization was analyzed by PlantmPLoc website (http://www.csbio.sjtu.edu.cn/bioinf/plant- multi/ ) [68, 69]. The *ARF* promoter sequences (i.e., selected as 2000 bp) were initially imported in Generic File Format (GFF) from the *L. chinense* genome. After that various cis-regulatory elements for each promoter sequence were identified by the PlantCARE database (http://bioinformatics.psb.ugent.be/webtools/plantcare/html/ ). The *L. chinense* genomic database was utilized for the chromosomal locations of *ARF* genes and was mapped based on available information. Protein-protein interaction (PPI) analyses of the LcARF family were performed on the STRING website (http://www.string-db.org ) to predict protein interactions. Moreover, we use Cytoscape (3.8.2) software to draw predicted protein network interaction maps.

### Phylogenetic analysis

Multiple sequence alignment (MSA) of ARF full-length proteins with FASTA format was done using MUSCLE (v3.8.31) set the following parameters: the maximum number of iterations was 1 with ‘-maxiters 1’ , and find diagonals with ‘-diags -sv -distance1 kbit20_3’. The trimmed MSA file was generated with trimAl (v1.4) set to ‘automated1’ mode and then used to construct the ARF phylogenetic tree. The Bayesian phylogenetic tree was constructed using the BEAST software (v2.6.6), by inputting the trimmed file in FASTA format with BEAUti 2 program [70].

### Somatic embryogenesis successive stages and various tissue transcriptomes of *L. chinense*

The immature embryos of *L. chinense* were used to induce embryogenic callus. After expanded culture on M13 (3/4MS + 30 g/L sucrose + 2 mg/L 2.4-D + 0.2 mg/L 6-BA) liquid medium, single cells were obtained by screening with a 400-mesh sieve and placed on 3/4MS (40 g/L sucrose + 0.8 g/L agar) medium to induce somatic embryogenesis. Embryogenic callus and five successive stages of somatic embryogenesis, i.e., globular embryo, heart-shaped embryo, torpedo embryo, early cotyledon embryo and cotyledon embryo, were collected and used for transcriptome sequencing. We used the transcriptomes of various tissues, i.e., shoot, leaf, bud, stigma, stamen, sepal, and petal. Among them, the induction of somatic embryogenesis was repeated three test cycles and callus and embryo samples were collected. Tissue samples were taken from 30-year-old *L. chinense* planted in Nanjing Forestry University. RNA-seq was used to sequence the RNA samples of tissues and somatic embryos, and then our genome sequence was used as a reference to draw clean readings. Trimmomatic (v0.36) was used to remove adaptors, poly- (A) tails, and low-quality reads from the original Illumina sequencing data. These clean reads were then matched to the SMRT long read reference sequence, and the number of matched reads for each reference sequence was calculated using RSEM (v1.3.0) to quantify the transcripts. The FPKM values of all mapped *LcARFs* are shown in Table S4 to calculate the transcript abundance of *LcARF* genes. The transcriptome data of this study have not been published.

### RNA extraction and qRT-PCR analysis

We collected samples of shoot, leaf, bud, stigma, stamen, sepal, petal and six embryonic development stages (callus, globular embryo, heart-shaped embryo, torpedo embryo, early cotyledon embryo, cotyledon embryo) of *L. chinense*. Tissue samples were taken from the adult trees of *L. chinense* in Nanjing Forestry University (Nanjing, China). Total RNA extraction was performed using a FastPure Total RNA Isolation Kit from Vazyme (Nanjing, China) (RC401) corporation. In this study, we set three biological replicates for each group of samples, and three technical replicates for each biological replicate [70]. All primers for qRT-PCR were designed by Primer3.0 and are listed in Table S5. All experiments were run on 96-well plates. All data generated from qRT-PCR were calculated by the 2^−△△CT^ formula. ACT97 was selected as the internal reference gene, one-way analysis of variance was performed using IBM SPSS Statistics 26, and GraphPad Prism 9 was used to draw the histogram.

### Subcellular localization

Using the predicted CDS sequences, we designed primers to clone the CDS sequences of six *LcARF* genes (*LcARF1/2a/5/9/16a/17a*) utilizing *L. chinense* cDNA as a template. Following this, we proceeded to clone the full-length coding sequences of *ARFs*, excluding stop codons, into the green fluorescent protein (GFP) vector (pCAMBIA1302) for subcellular localization. The vector construction and primer information are detailed in the Table S5. The recombinant plasmid and the vector with nuclear localization signal (35S::H2B::Mcherry) were transiently transformed into protoplasts of *Liriodendron* callus by PEG-mediated transformation [71]. After overnight incubation at 23 °C in the dark, a Zeiss LSM 480 fluorescence confocal microscopy for detecting GFP and M cherry signal.

### Obtaining genetically modified *Liriodendron*

To obtain transgenic *Liriodendron* callus, we first selected *Liriodendron* callus with a good growth state (fine particles, uniform size, light yellow color) as the experimental material. Then, we used the plant binary expression vectors (*35S::LcARF1::GFP*, *35S::LcARF2a::GFP*, *35S::LcARF5::GFP* ) constructed in 4.6 and introduced the vector into callus cells by Agrobacterium-mediated transformation [8]. Through this process, transgenic lines were obtained. We used ACT97 as an internal reference gene, and used qRT-PCR experiments to screen transgenic lines with overexpression of the target gene. The specific primers used in the experiment are listed in Table S5.

### Detection of callus activity of transgenic lines

The callus from the transgenic lines was carefully rinsed with phosphate buffer (0.1M, pH = 8.0) and placed onto a slide. The cell morphology was observed under an inverted microscope. Using the ImageJ software, the cells' length and width were measured, and the cell aspect ratio was calculated. At least 100 cells were measured randomly for each line. Acetic acid magenta and Evans blue staining were used to assess callus cell activity further. Acetic acid magenta is capable of staining chromosomes or chromatin into a shade of purplish-red, facilitating the observation of cell mitosis. In an acidic environment, the carboxyl group of magenta acetate can become charged, allowing it to polarly bind to structural components such as chromosomes and nuclei, thereby staining them. Typically, darker staining indicates higher cell viability. On the other hand, Evans blue is capable of binding to proteins to form Evans blue protein complexes, which cannot penetrate normal cell membranes. However, when cells are damaged, Evans blue can penetrate the membrane and bind to proteins, turning them blue. Thus, deeper staining indicates more damaged cells. The calli from the transgenic lines were treated with stains and washed thrice with PBS after 10 min. The stained calli were then observed using a stereomicroscope.

### Induction and observation of somatic embryogenesis

Suspension cultured calluses were screened using 150-mesh and 400-mesh sieves. The single cells on the 400 mesh sieve were rinsed with 3/4MS liquid medium and placed on 3/4MS medium supplemented with agar (0.8 g/L) to induce somatic embryogenesis [5]. The globular embryos, torpedo embryos and cotyledon embryos of the experimental group (*pLcARF1::GFP*) and the control group (*35S::GFP*) were selected to observe the distribution of GFP fluorescence signals in embryos.

### Supplementary Information

**Additional file 1: Table S1.** *LcARFs* position of Chromosome.

**Additional file 2: Table S2.** Evolutionary tree of LcARF.

**Additional file 3: Table S3.** List of domains, MR, CDS, cis-regulatory elements information of the identified LcARF.

**Additional file 4: Table S4.** FPKM values for different tissue and somatic embryogenesis processes in *LcARF*.

**Additional file 5: Table S5.** List of primers.

**Additional file 6: Figure S1.** Genomic distribution of *LcARF* genes on chromosomes.

**Additional file 7: Figure S2.** Classification of *Liriodendron chinense* ARF proteins.

**Additional file 8: Figure S3.** qRT-PCR was used to detect the overexpression of target genes in the transgenic callus.

## Data Availability

All data analyzed during this study are included in this article and its additional files.

## Abbreviations

qRT-PCR: Quantitative real-time reverse transcription PCR
IAA: Indole Acetic Acid
ABA: Abscisic Acid
MeJA: Methyl Jasmonate
