# Supplementary figures and images for: Genomic survey and expression analysis of LcARFs reveal multiple functions to somatic embryogenesis in Liriodendron
Source: BMC Plant Biol. 2024 Feb 7;24:94. doi: 10.1186/s12870-024-04765-7 (PMC10848544; doi:10.1186/s12870-024-04765-7)

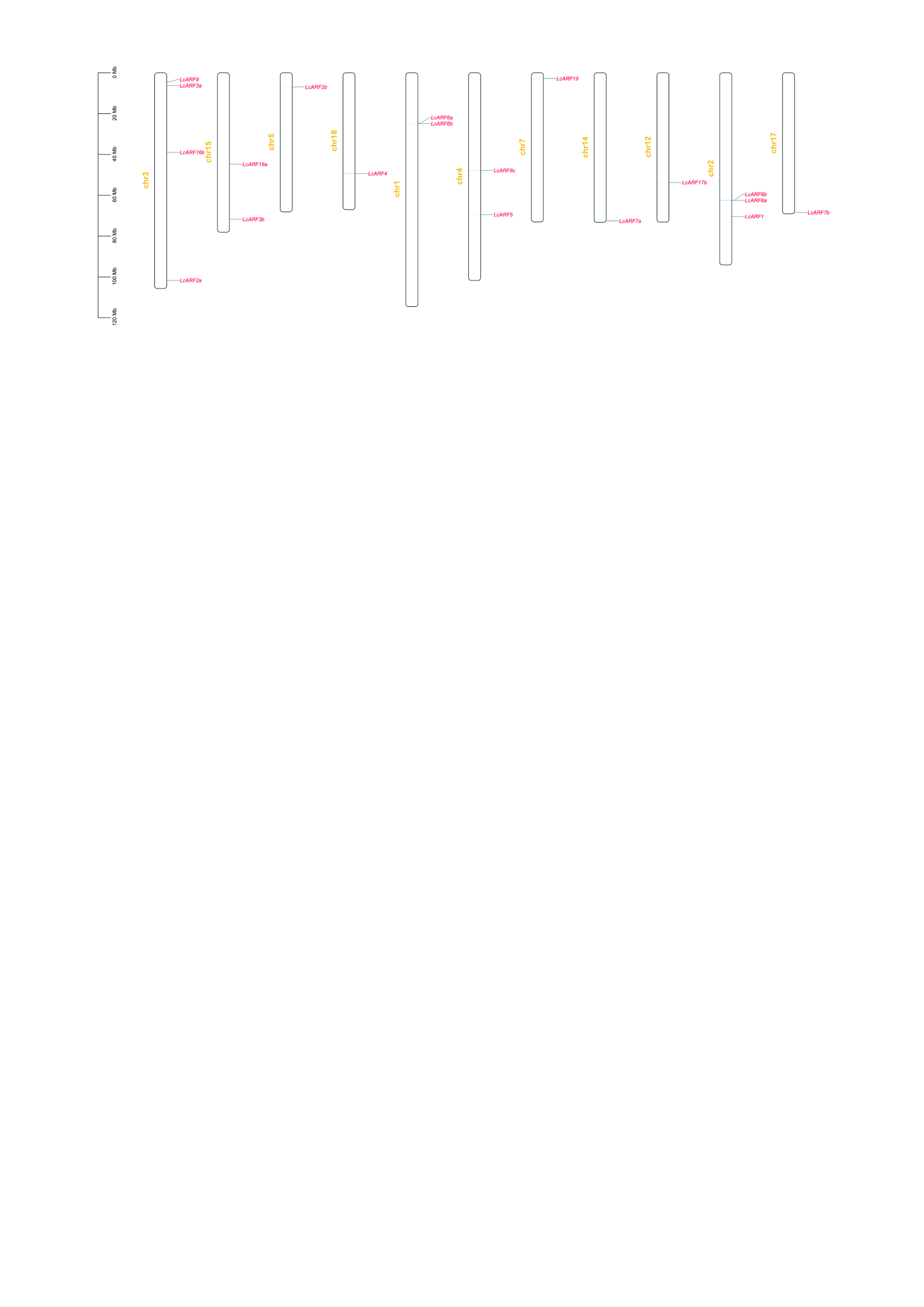

Supplement: Supplementary file 6 — Additional file 6: Figure S1. Genomic distribution of LcARF genes on chromosomes. [file 12870_2024_4765_MOESM6_ESM.tif]

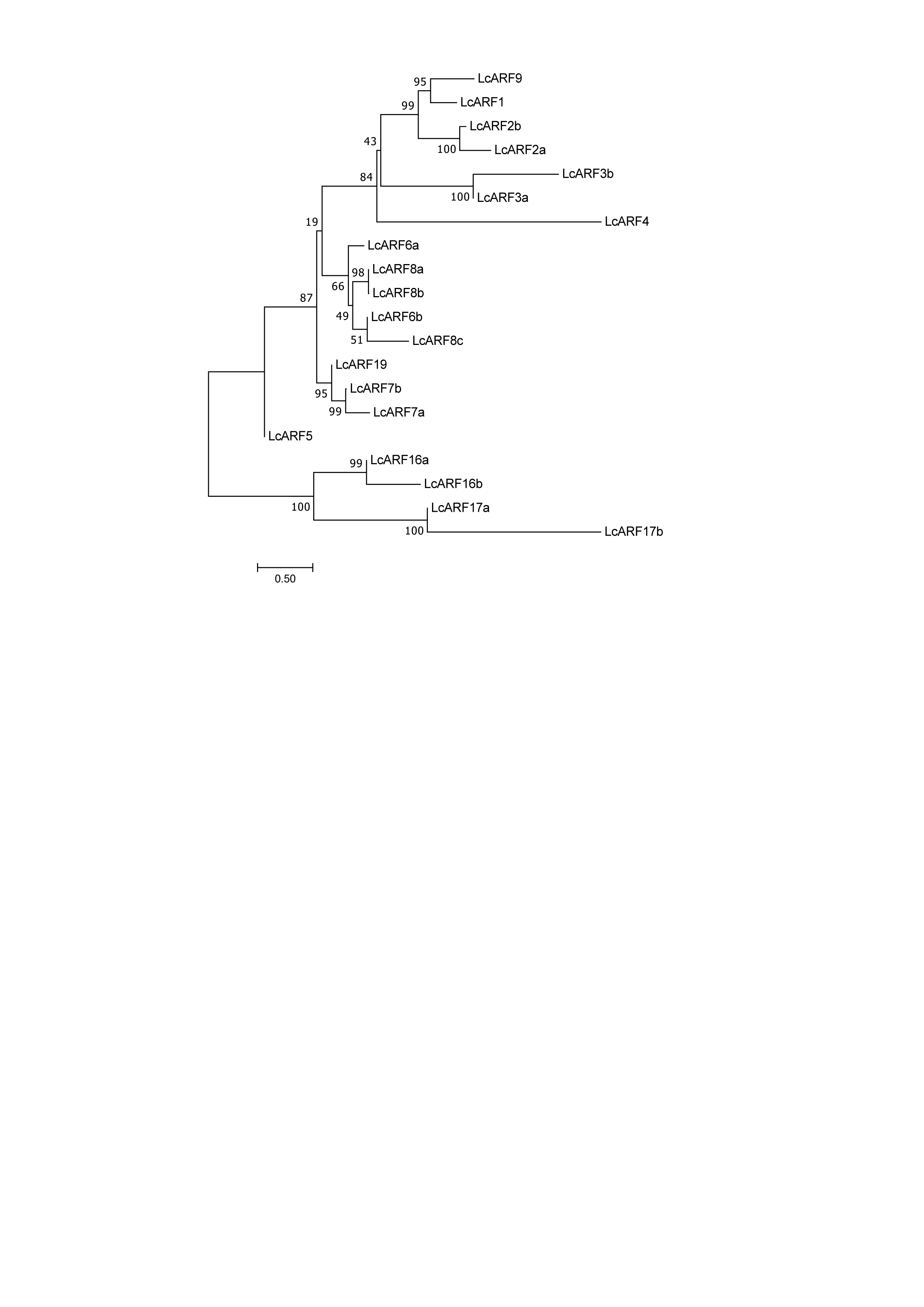

Supplement: Supplementary file 7 — Additional file 7: Figure S2. Classification of Liriodendron chinense ARF proteins. [file 12870_2024_4765_MOESM7_ESM.tif]

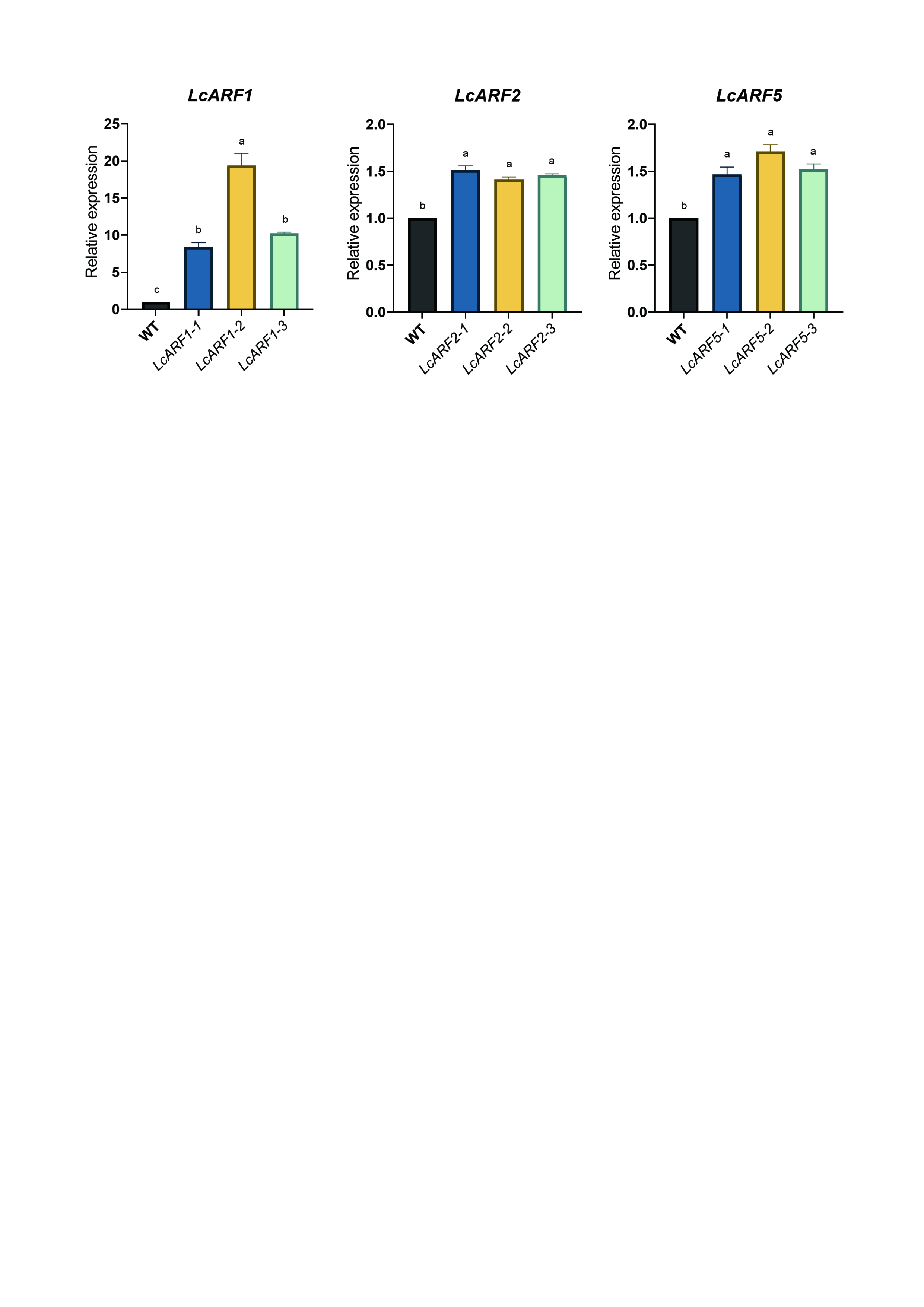

Supplement: Supplementary file 8 — Additional file 8: Figure S3. qRT-PCR was used to detect the overexpression of target genes in the transgenic callus. [file 12870_2024_4765_MOESM8_ESM.tif]
